# Development of a Minimum Data Set (MDS) for the National Suicide Registry, India

**DOI:** 10.7759/cureus.89195

**Published:** 2025-08-01

**Authors:** Karthi Vignesh Raj K, Ramdas Ransing, Ramalho Rodrigo, Nilesh Devraj

**Affiliations:** 1 Forensic Medicine and Toxicology, All India Institute of Medical Sciences, Guwahati, Guwahati, IND; 2 Psychiatry, All India Institute of Medical Sciences, Guwahati, Guwahati, IND; 3 Department of Social and Community Health, Faculty of Medical and Health Sciences, University of Auckland, Auckland, NZL; 4 Forensic Medicine, All India Institute of Medical Sciences, Guwahati, Guwahati, IND

**Keywords:** data collection, data elements, registry, risk factors, suicide

## Abstract

Background: Suicide is a major health concern around the globe, including India. Identifying various risk factors and confirming individual cases of is challenging to investigating agencies and clinicians. This study was carried out with the intention of preparing a standardized minimum data set (MDS) for the National Suicide Registry of India (NSRI) as recommended by the National Suicide Prevention Strategy (NSPS) for framing policy decisions on suicide.

Methods:This study was conducted in three steps: a literature review to identify data elements, appointing experts and finalization of MDS items and content validation by two rounds of Delphi. The data elements were validated by calculating content validity index (I-CVI) and content validity ratio (CVR).

Result: After literature review, the extracted data elements were divided into eight headings and forwarded to multidisciplinary experts. A total of 28 experts participated in this study. A total of 176 data elements were evaluated, out of which 169 data elements were finalized based upon I-CVI and CVR calculation and used for standardizing this MDS.

Conclusion: A standardized MDS has been developed for NSRI. This MDS will facilitate uniform data collection on suicides across the country, addressing existing limitations in data collection. By analyzing data gathered through the MDS, policymakers can inform the NSPS, ultimately helping to reduce suicide rates in India.

## Introduction

The effect of a disease, condition, or exposure on an individual and its adverse health effects can be identified through the systematic gathering of its data, known as a health registry [1,2]. Through collaboration and integration with multiple health stakeholders, a health registry not only increases medical knowledge about a disease but also assists individuals, communities, or nations in understanding the specifics of a disease and improving their health. The three essential stakeholders or pillars for establishing a health register are medical specialists, political will for approval, and management or administrative stakeholders to ensure the registry's effectiveness [3-5].

Suicide is a growing public health problem around the world, including India. Suicide is a death resulting from intentional use of physical force or power against oneself, with a preponderance of evidence indicating that the use of force was intentional [6]. Multidimensional factors such as financial, social, academic, health, and environmental difficulties influence suicide attempts or suicide. Globally, ingestion of poison, hanging and firearms are the most common methods of committing suicide, and suicide attempts are the most important risk factors for suicide [7,8]. India and China account for 49% out of 84% of global suicides that occur in low- and middle-income countries [9]. As per the recent data published by the National Crime Records Bureau (NCRB) of India, India's suicide rate per 100,000 population was 11.3 in 2020, 12.1 in 2021, and 12.4 in 2022, which is higher than the global average [10-12]. Every year, more than seven lakh people die by suicide. Although suicide and self-harm are common in India, the NCRB data represents only the tip of the iceberg; suicides are often underreported in India [13]. Suicide deaths in India are underreported by 20-25% and may be as much as six to nine times higher than the "official" rate reported by the NCRB [14]. NCRB data has several flaws, such as a lack of uniform classification of suicide deaths and the absence of real-time data collection, which limits its utility for developing suicide prevention policies [15]. To the best of our knowledge, India lacks a reliable hospital autopsy-based register and surveillance system for gathering and tracking data on suicide. The forensic medicine and toxicology departments of medical institutions can not only assist in accurately identifying suicide cases through post-mortem examinations, as seen during the COVID-19 pandemic [16,17], but also in distinguishing these cases from others that may be misclassified or misinterpreted as suicide cases by NCRB officials, thereby hindering policy-making decisions. Thus, the aim of the study was to create a hospital autopsy-based registry, with the objective that its adoption will help with the development of an effective monitoring mechanism to estimate the suicide rate and identify risk factors and methods of suicide in India.

## Materials and methods

**Figure 1:**
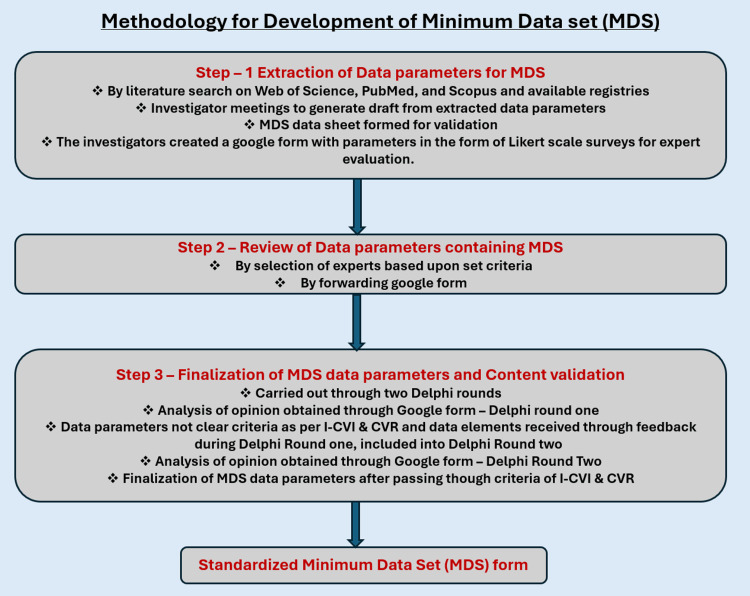
Methodology Flow Diagram Acronyms: MDS = Minimum Data Set, Content Validity Ratio = CVR, Index - Content Validity Index = I-CVI

The following method was used to prepare the minimum data set (MDS) for the hospital autopsy-based suicide registry (Figure 1) [16]. 

First step: comprehensive literature search and preparation of drafts of the MDS

A team of investigators carried out a comprehensive literature search to define and identify the MDS items. The databases (Web of Science, PubMed, and Scopus) and available registries were searched for the identification of MDS items. The literature and registry search were continued until item saturation was reached. The literature was in English and published from the year of 2000 to 2025. Several meetings were held among investigators to generate drafts of the MDS data elements, after which the preliminary MDS data collection sheet was finalized for expert review. The preliminary MDS datasheet consisted of two parts, namely, the administrative data and the clinical/post-mortem data. The datasheet included a closed questionnaire with different types of questions, including multiple- and single-choice questions, open-text questions for residency, date, and time.

Second step: expert panel for review of the MDS

The inclusion criteria for experts were 1) professionals from related disciplines (forensic medicine and toxicology, psychiatry, psychologist, social work, community/public health, medicine, surgery, and emergency medicine), 2) having more than three years of experience working on suicide, 3) willingness to participate and return the answers to the researchers, 4) consistent participation in both rounds of the Delphi process for the finalization of the MDS.

Third step: finalization of MDS items and content validation

The expert panel reviewed the preliminary MDS elements in two iterative rounds, via Google Forms (Alphabet Inc., Mountain View, CA, USA) without face-to-face contact, and opinion was obtained. Content validation index (I-CVI) and content validation ratio (CVR) were used for validating the data elements [17,18]. Data elements with acceptable validity were compared with the Lawshe table value and included in the final MDS [19]. The data elements on which questionnaires were developed for Delphi round one and two are attached in the appendices.

Study tools

The following tools were used in this study: 1) Standardized Likert scale method and Delphi method used to obtain opinions from experts [20,21]; 2) Item ratings for I-CVI: A five-point ordinal scale (as per standard guidelines) was used for item ratings with neutral opinion. The rating was done using following options 1) strongly disagree 2) disagree 3) neutral 4) agree 5) strongly agree; 3) Item ratings for CVR: The item ratings of CVR were on a five-point ordinal scale for the criterion necessity (1 = essential, 2 = useful, 3 = neutral, 4 = not essential, and 5 = not useful).

Sample size

Selection of a Panel of Experts

In the Delphi method, there is no specific method for determining the sample size; however, a minimum of 10 to 15 participants is generally considered sufficient for consensus. Delphi sample sizes rely more on group dynamics in reaching consensus than on statistical power [22]. In this study, a heterogeneous sample of experts was purposively selected, all of whom had experience working with people who died by suicide. Considering a potential 20% drop-out rate per Delphi round, a minimum of 40 experts from different backgrounds were recruited to reduce the error rate [23], out of which 28 experts participated in the first and second steps of Delphi rounds.

Data analysis for the first round

The experts were asked to provide an opinion on each data element to be included in the MDS using a five-point Likert scale. Participant responses were anonymous throughout the survey. Experts were also invited to propose new items not included in the preliminary MDS for subsequent prioritization. The validity of the questionnaire was evaluated by the team of investigators using the CVR and I-CVI.

The formula for Content Validity Ratio is CVR = (Ns - N/2)/(N/2), where Ns represents the total number of specialists/evaluators giving a rating of 4 or 5 to extracted data elements and N represents the total number of specialists/evaluators.

The formula for Index - Content Validity Index is I-CVI = Ns/N, where Ns represents the total number of specialists/evaluators giving a rating of 4 or 5 to extracted data elements and N represents the total number of specialists/evaluators. 

For each data parameter, I-CVI was calculated and data parameters with an I-CVI >0.75 and a CVR >0.33 were kept. Items with an I-CVI <0.75 and a CVR <0.33 were moved to the second round of the Delphi process.

Data analysis for the second round

Data elements with an I-CVI < 0.75 and a CVR < 0.33 were re-evaluated in the second round of the Delphi process. Experts rated these items on a five-point Likert scale ranging from 1 (very low importance) to 5 (very high importance), along with suggestions and feedback received during from the first round. Proposed revisions were shared again with the experts for their opinion on a Likert scale. Items with a CVR ≥0.33 and an I-CVI ≥0.75 were kept in the final MDS. Items with a CVR below the acceptable threshold were removed due to insufficient validity. 

## Results

In this study, we searched various databases to obtain relevant data elements and circulated these among experts for validation using a two-round Delphi survey. A Google Form containing all the extracted data elements and item ratings was created for expert validation. Individual emails with the Google Form link were sent to all experts to gather unbiased opinions. The email included a formal introduction to the development of an MDS for suicide studies, instructions for the Delphi process to validate the data elements, and the Google Form link. Follow-up reminder emails or phone calls were made to experts who had not responded.

**Figure 2:**
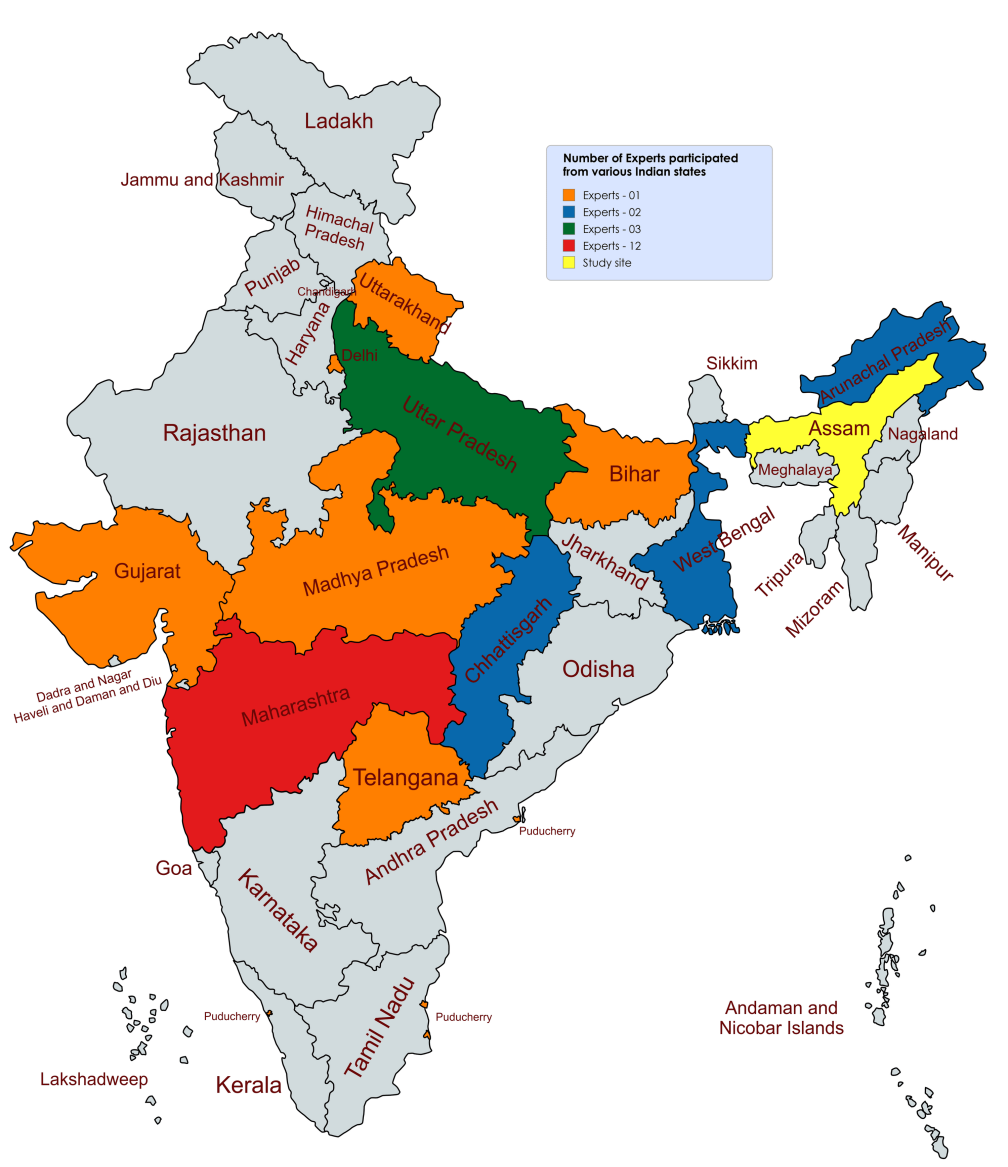
Number of experts who participated from Indian states Total number of experts (28) = One expert from each state in orange (Uttarakhand, Delhi, Bihar, Madhya Pradesh, Gujrat, Telangana and Puducherry [as one single state]; N = 7), two experts from each state in blue (Chhattisgarh and West Bengal; N = 6), three experts from Uttar Pradesh in green (N = 3), and 12 experts from Maharashtra in red (N = 12).

We included a minimum of 40 expert participants, accounting for possible dropouts or lack of feedback. A total of 28 experts (Figure 2) from various states of India participated and validated all data elements in both Delphi stages.

We selected these experts based on their research on suicide and years of experience: 1) less than five years, 2) more than five but less than 10 years, and 3) more than 10 years. Feedback was received from professionals with more than five years of expertise; no expert had less than five years of experience. In both rounds, responses were received from all 28 experts; 24 (85.71%) had over 10 years of experience, while four (14.28%) had between five and 10 years. The experts represented diverse specialties, including four in community and family medicine, 17 in forensic medicine and toxicology, four as intensivists/physicians, one in psychiatry and two psychologists. Of the 28 experts, three (10.71%) were female and 25 (89.28%) were male. The overall expert response rate was 70%.

The extracted data elements (Table 1) were under seven main headings, which included 1) Deceased profile (with two sub-headings), 2) Religion, relationship and residence status (with four sub-headings), 3) Socio-economic status as per Modified Kuppuswamy Scale [24] (with four sub-headings), 4) History obtained from relative/investigating agency (with two sub-headings), 5) Elements to evaluate the reason behind the current suicide (with 10 sub-headings), 6) Clinical and psychological factors (with four sub-headings), 7) Suicide method (with five sub-headings). During the first round of Delphi, eight data elements did not qualify for set norms and few experts provided their expertise feedback under each heading. All unqualified elements, along with data elements received through feedback, were included in the second round of Delphi.

**Table 1:** Extracted data elements for Delphi Phase round 1 Acronyms: Content Validity Index = I-CVI, Content Validity Ratio = CVR, HRA: Housing Rental Allowance

Delphi Phase												
Round 1												
Sr. No.	Extracted data elements	Disagree (1+2)		Neutral (3)		Agree (4+5)		Total number of responses		I-CVI- Round 1	Decision	CVR – Round 1
		count	%	count	%	count	%	count	%			
1	Deceased Profile											
a	Gender											
i	Male	0	0.00	0	0.00	28	100.00	28	100.00	1.00	kept	1
ii	Female	0	0.00	0	0.00	28	100.00	28	100.00	1.00	kept	1
iii	Transgender	1	3.57	2	7.14	25	89.29	28	100.00	0.89	kept	0.7857143
iv	Gender [Any other (conversion of gender)]	6	21.43	6	21.43	16	57.14	28	100.00	0.57	moved for 2nd round	0.1428571
v	Gender [Don't want to disclose]	11	39.29	7	25.00	10	35.71	28	100.00	0.36	moved for 2nd round	-0.2857143
b	Age											
i	Age [Based upon Date of Birth (DOB) (if Known)]	1	3.57	2	7.14	25	89.29	28	100.00	0.89	kept	0.7857143
ii	Age [If DOB not known, then as reported on any document]	1	3.57	2	7.14	25	89.29	28	100.00	0.89	kept	0.7857143
iii	Age [If DOB not known or no document available, then as informed by relative or police]	2	7.14	5	17.86	21	75.00	28	100.00	0.75	kept	0.5
2	Religion, Relationship and Residence Status											
a	Religion											
i	Hindu	2	7.14	4	14.29	22	78.57	28	100.00	0.79	kept	0.5714286
ii	Muslim	2	7.14	5	17.86	21	75.00	28	100.00	0.75	kept	0.5
iii	Christian	2	7.14	5	17.86	21	75.00	28	100.00	0.75	kept	0.5
iv	Sikh	2	7.14	5	17.86	21	75.00	28	100.00	0.75	kept	0.5
v	Buddhist	2	7.14	5	17.86	21	75.00	28	100.00	0.75	kept	0.5
vi	Jain	2	7.14	5	17.86	21	75.00	28	100.00	0.75	kept	0.5
vii	Any other	2	7.14	5	17.86	21	75.00	28	100.00	0.75	kept	0.5
viii	Don’t want to disclose	6	21.43	9	32.14	13	46.43	28	100.00	0.46	moved for 2nd round	-0.0714286
b	Marital/ Relationship Status											
i	Never married	1	3.57	2	7.14	25	89.29	28	100.00	0.89	kept	0.7857143
ii	Widowed	1	3.57	2	7.14	25	89.29	28	100.00	0.89	kept	0.7857143
iii	Divorced	1	3.57	2	7.14	25	89.29	28	100.00	0.89	kept	0.7857143
iv	Separated	1	3.57	4	14.29	23	82.14	28	100.00	0.82	kept	0.6428571
v	Married	0	0.00	1	3.57	27	96.43	28	100.00	0.96	kept	0.9285714
vi	Not stated	7	25.00	8	28.57	13	46.43	28	100.00	0.46	moved for 2nd round	-0.0714286
vii	Live in relationship (emotional attachment)	0	0.00	2	7.14	26	92.86	28	100.00	0.93	kept	0.8571429
c	Family Structure											
i	Proton	1	3.57	1	3.57	26	92.86	28	100.00	0.93	kept	0.8571429
ii	Electron	2	7.14	2	7.14	24	85.71	28	100.00	0.86	kept	0.7142857
iii	Nuclear	1	3.57	1	3.57	26	92.86	28	100.00	0.93	kept	0.8571429
vi	Atom	2	7.41	1	3.70	24	88.89	27	100.00	0.89	kept	0.7142857
v	Molecular	4	14.29	1	3.57	23	82.14	28	100.00	0.82	kept	0.6428571
vi	Joint	3	10.71	1	3.57	24	85.71	28	100.00	0.86	kept	0.7142857
vii	Quasi	3	10.71	2	7.14	23	82.14	28	100.00	0.82	kept	0.6428571
d	Residence											
i	Based upon HRA	3	10.71	3	10.71	22	78.57	28	100.00	0.79	kept	0.5714286
ii	Based upon Population	0	0.00	5	17.86	23	82.14	28	100.00	0.82	kept	0.6428571
e	Type of Residence											
i	House/Town home	0	0.00	7	25.00	21	75.00	28	100.00	0.75	kept	0.5
ii	Apartment	0	0.00	6	21.43	22	78.57	28	100.00	0.79	kept	0.5714286
iii	Homeless	2	7.14	2	7.14	24	85.71	28	100.00	0.86	kept	0.7142857
iv	Treatment facility	0	0.00	1	3.57	27	96.43	28	100.00	0.96	kept	0.9285714
v	Correction facility	0	0.00	2	7.14	26	92.86	28	100.00	0.93	kept	0.8571429
vi	Unknown	3	10.71	6	21.43	19	67.86	28	100.00	0.68	moved for 2nd round	0.3571429
vii	Other, specify	0	0.00	8	28.57	20	71.43	28	100.00	0.71	moved for 2nd round	0.4285714
f	Deceased residing with											
i	Parents	2	7.14	3	10.71	23	82.14	28	100.00	0.82	kept	0.6428571
ii	Spouse	1	3.57	2	7.14	25	89.29	28	100.00	0.89	kept	0.7857143
iii	Room mates	2	7.14	3	10.71	23	82.14	28	100.00	0.82	kept	0.6428571
iv	Children	2	7.14	3	10.71	23	82.14	28	100.00	0.82	kept	0.6428571
v	No one residing	1	3.57	4	14.29	23	82.14	28	100.00	0.82	kept	0.6428571
vi	Unknown	3	10.71	6	21.43	19	67.86	28	100.00	0.68	moved for 2nd round	0.3571429
vii	Other, specify	1	3.57	7	25.00	20	71.43	28	100.00	0.71	moved for 2nd round	0.4285714
g	Recent resident problem											
i	Recent eviction	1	3.57	2	7.14	25	89.29	28	100.00	0.89	kept	0.7857143
ii	Threat for eviction	1	3.57	2	7.14	25	89.29	28	100.00	0.89	kept	0.7857143
iii	Recent foreclosure	1	3.57	3	10.71	24	85.71	28	100.00	0.86	kept	0.7142857
iv	Threat foreclosure	1	3.57	2	7.14	25	89.29	28	100.00	0.89	kept	0.7857143
3	Socio-economic status as per Modified Kuppuswamy Scale [24]											
A	Occupation - by Kuppuswamy scale	1	3.57	3	10.71	24	85.71	28	100.00	0.86	kept	0.7142857
B	Education - by Kuppuswamy scale	1	3.57	2	7.14	25	89.29	28	100.00	0.89	kept	0.7857143
C	Income - by Kuppuswamy scale	2	7.14	2	7.14	24	85.71	28	100.00	0.86	kept	0.7142857
d	Socio-economic class - by Kuppuswamy scale	1	3.57	4	14.29	23	82.14	28	100.00	0.82	kept	0.6428571
4	History Obtained from Relative/Investigating Agency											
a	Who saw the body 1st time or found it?											
i	Family members -1st degree	0	0.00	5	17.24	24	82.76	29	100.00	0.83	kept	0.7142857
ii	Family members - other than1st degree	0	0.00	6	21.43	22	78.57	28	100.00	0.79	kept	0.5714286
iii	Friends	0	0.00	7	25.00	21	75.00	28	100.00	0.75	kept	0.5
iv	Neighbours	0	0.00	6	21.43	22	78.57	28	100.00	0.79	kept	0.5714286
v	Police	1	3.57	6	21.43	21	75.00	28	100.00	0.75	kept	0.5
vi	Social worker/NGO	1	3.57	6	21.43	21	75.00	28	100.00	0.75	kept	0.5
vii	Others	1	3.57	6	21.43	21	75.00	28	100.00	0.75	kept	0.5
b	Location of the incidence											
i	Own residence	1	3.57	3	10.71	24	85.71	28	100.00	0.86	kept	0.7142857
ii	Rented house	1	3.57	4	14.29	23	82.14	28	100.00	0.82	kept	0.6428571
iii	Forest area	2	7.14	3	10.71	23	82.14	28	100.00	0.82	kept	0.6428571
iv	Farm	1	3.57	3	10.71	24	85.71	28	100.00	0.86	kept	0.7142857
v	At workplace	2	7.14	3	10.71	23	82.14	28	100.00	0.82	kept	0.6428571
vi	Hostel	1	3.57	2	7.14	25	89.29	28	100.00	0.89	kept	0.7857143
vii	School	2	7.14	3	10.71	23	82.14	28	100.00	0.82	kept	0.6428571
viii	Police lockup	1	3.57	3	10.71	24	85.71	28	100.00	0.86	kept	0.7142857
ix	Prison	1	3.57	3	10.71	24	85.71	28	100.00	0.86	kept	0.7142857
x	Construction site	2	7.14	3	10.71	23	82.14	28	100.00	0.82	kept	0.6428571
xi	Old age home	2	7.14	3	10.71	23	82.14	28	100.00	0.82	kept	0.6428571
xii	Other, specify	1	3.57	5	17.86	22	78.57	28	100.00	0.79	kept	0.5714286
5	Elements to evaluate the reason behind the current suicide											
a	Is there any isolation during the attempt?	1	3.57	1	3.57	26	92.86	28	100.00	0.93	kept	0.8571429
b	Material or object used for suicide, is it already available at crime scene?	0	0.00	0	0.00	28	100.00	28	100.00	1.00	kept	1
c	If answer for above question is “No”, then material or object is purchased or brought from somewhere outside?	3	10.71	3	10.71	22	78.57	28	100.00	0.79	kept	0.5714286
d	Is there suicide note found on the scene?	0	0.00	2	7.14	26	92.86	28	100.00	0.93	kept	0.8571429
e	Does deceased seek any help during the attempt?	0	0.00	2	7.14	26	92.86	28	100.00	0.93	kept	0.8571429
f	Is there any clear expression of suicide intention?	0	0.00	2	7.14	26	92.86	28	100.00	0.93	kept	0.8571429
g	Alleged purpose of suicide attempt?	0	0.00	2	7.14	26	92.86	28	100.00	0.93	kept	0.8571429
h	The number of suicidal ideation/attempts.	0	0.00	2	7.14	26	92.86	28	100.00	0.93	kept	0.8571429
i	Family issues											
i	Family conflict	0	0.00	0	0.00	28	100.00	28	100.00	1.00	kept	1
ii	Peer conflict	0	0.00	2	7.14	26	92.86	28	100.00	0.93	kept	0.8571429
iii	Spouse problems/ Marital-partner relationship difficulties	0	0.00	1	3.57	27	96.43	28	100.00	0.96	kept	0.9285714
iv	Relationship breakdown with an intimate partner (past 1 month)	0	0.00	1	3.57	27	96.43	28	100.00	0.96	kept	0.9285714
v	Some legal issues or dispute	0	0.00	2	7.14	26	92.86	28	100.00	0.93	kept	0.8571429
vi	Death of a close family member	1	3.57	1	3.57	26	92.86	28	100.00	0.93	kept	0.8571429
vii	Parent separation	0	0.00	3	10.71	25	89.29	28	100.00	0.89	kept	0.7857143
viii	Issue with Position in the household – head, child, wife, unknown	1	3.57	2	7.14	25	89.29	28	100.00	0.89	kept	0.7857143
j	Social issues											
i	Social and teamwork activities – a) very minimum b) minimum c) moderate d) much	1	3.57	1	3.57	26	92.86	28	100.00	0.93	kept	0.8571429
ii	Antisocial activities	1	3.57	1	3.57	26	92.86	28	100.00	0.93	kept	0.8571429
iii	Work problems	1	3.57	0	0.00	27	96.43	28	100.00	0.96	kept	0.9285714
iv	Recent job loss	1	3.57	1	3.57	26	92.86	28	100.00	0.93	kept	0.8571429
v	Issues related to Education like fail in exam, exam grade, suspension or ragging	0	0.00	2	7.14	26	92.86	28	100.00	0.93	kept	0.8571429
vi	Victim of domestic violence	0	0.00	1	3.57	27	96.43	28	100.00	0.96	kept	0.9285714
	Clinical and psychological factors											
a	History of medical problems											
i	Diagnosed communicable illness such as HIV/TB/HBV/HCV	0	0.00	1	3.57	27	96.43	28	100.00	0.96	kept	0.9285714
ii	Suffering from non-communicable illness, any chronic illness/condition (e.g., cancer)	0	0.00	1	3.57	27	96.43	28	100.00	0.96	kept	0.9285714
iii	Recent serious injury	0	0.00	2	7.14	26	92.86	28	100.00	0.93	kept	0.8571429
b	History of mental disorders (e.g., delusion, mood, personality & anxiety disorder, schizophrenia or any other disorder, sleep disorder)	0	0.00	0	0.00	28	100.00	28	100.00	1.00	kept	1
c	History of drug Addiction											
i	Alcohol dependence	0	0.00	1	3.57	27	96.43	28	100.00	0.96	kept	0.9285714
ii	Chronic smoker/Nicotine addict	0	0.00	1	3.57	27	96.43	28	100.00	0.96	kept	0.9285714
iii	Opioid dependence	0	0.00	1	3.57	27	96.43	28	100.00	0.96	kept	0.9285714
iv	Cannabis dependence	0	0.00	2	7.14	26	92.86	28	100.00	0.93	kept	0.8571429
v	Any other drug dependence	0	0.00	2	7.14	26	92.86	28	100.00	0.93	kept	0.8571429
d	Period during which suicide committed											
i	00.00am – 06.00Hrs	1	3.57	6	21.43	21	75.00	28	100.00	0.75	kept	0.5
ii	06.01hrs – 12.00Hrs	1	3.57	6	21.43	21	75.00	28	100.00	0.75	kept	0.5
iii	12.01Hrs – 18.00Hrs	1	3.57	6	21.43	21	75.00	28	100.00	0.75	kept	0.5
iv	18.01Hrs – 23.59Hrs	1	3.57	6	21.43	21	75.00	28	100.00	0.75	kept	0.5
	Suicide Method											
a	Probable cause of death as reported by Police before PM examination											
i	Hanging	1	3.57	1	3.57	26	92.86	28	100.00	0.93	kept	0.8571429
ii	Accidental strangulation	3	10.71	4	14.29	21	75.00	28	100.00	0.75	kept	0.5
iii	Suffocation	2	7.14	3	10.71	23	82.14	28	100.00	0.82	kept	0.6428571
iv	Insecticide consumption	1	3.57	1	3.57	26	92.86	28	100.00	0.93	kept	0.8571429
v	Consumption of other poison/ poison which is not known	1	3.57	2	7.14	25	89.29	28	100.00	0.89	kept	0.7857143
vi	Jumping (from height, in front of train or running vehicle)	1	3.57	3	10.71	24	85.71	28	100.00	0.86	kept	0.7142857
vii	By setting fire with the use of inflammable agent	1	3.57	3	10.71	24	85.71	28	100.00	0.86	kept	0.7142857
viii	By using Firearm weapon or by explosive material	2	7.14	2	7.14	24	85.71	28	100.00	0.86	kept	0.7142857
ix	By using sharp object	2	7.14	2	7.14	24	85.71	28	100.00	0.86	kept	0.7142857
x	By using blunt object	4	14.29	2	7.14	22	78.57	28	100.00	0.79	kept	0.5714286
xi	By use of electrocution	2	7.41	3	11.11	22	81.48	27	100.00	0.81	kept	0.5714286
xii	Other, please specify	3	10.71	3	10.71	22	78.57	28	100.00	0.79	kept	0.5714286
b	Suicide method- By self-harm											
i	Hanging/strangulation/ suffocation	0	0.00	0	0.00	28	100.00	28	100.00	1.00	kept	1
ii	Drowning	1	3.57	0	0.00	27	96.43	28	100.00	0.96	kept	0.9285714
iii	Pesticide (insecticide) consumption	0	0.00	0	0.00	28	100.00	28	100.00	1.00	kept	1
iv	Consumption of other poison	0	0.00	0	0.00	28	100.00	28	100.00	1.00	kept	1
v	Jumping (from height, in front of train or running vehicle)	0	0.00	2	7.14	26	92.86	28	100.00	0.93	kept	0.8571429
vi	Any other method which is not included above	0	0.00	2	7.14	26	92.86	28	100.00	0.93	kept	0.8571429
vii	By setting fire with the use of inflammable agent	1	3.57	1	3.57	26	92.86	28	100.00	0.93	kept	0.8571429
viii	By using Firearm weapon or by explosive material	0	0.00	2	7.14	26	92.86	28	100.00	0.93	kept	0.8571429
ix	By using sharp object	0	0.00	1	3.57	27	96.43	28	100.00	0.96	kept	0.9285714
x	By using blunt object	1	3.57	2	7.14	25	89.29	28	100.00	0.89	kept	0.7857143
xi	By use of electrocution	0	0.00	3	10.71	25	89.29	28	100.00	0.89	kept	0.7857143
xii	Other, please specify	2	7.14	3	10.71	23	82.14	28	100.00	0.82	kept	0.6428571
c	Suicide Method - By self-poisoning											
i	Overdose of drug by oral ingestion	0	0.00	1	3.57	27	96.43	28	100.00	0.96	kept	0.9285714
ii	Overdose of drug by i.v. or i.m. by other routes	0	0.00	2	7.14	26	92.86	28	100.00	0.93	kept	0.8571429
iii	Overdose of alcohol	0	0.00	2	7.14	26	92.86	28	100.00	0.93	kept	0.8571429
iv	Exposure to organic solvents or hydrogenated hydrocarbons and their vapours	1	3.57	1	3.57	26	92.86	28	100.00	0.93	kept	0.8571429
v	Exposure to asphyxiant toxic gases	1	3.57	2	7.14	25	89.29	28	100.00	0.89	kept	0.7857143
vi	Exposure to pesticide	0	0.00	2	7.14	26	92.86	28	100.00	0.93	kept	0.8571429
vii	Exposure to unspecified chemicals or poison	1	3.57	2	7.14	25	89.29	28	100.00	0.89	kept	0.7857143
viii	Other, please specify………	1	3.57	2	7.14	25	89.29	28	100.00	0.89	kept	0.7857143
d	Number of suicide method used to commit suicide, which was confirmed after PME											
i	Single method used	0	0.00	2	7.14	26	92.86	28	100.00	0.93	kept	0.8571429
ii	Double method used	0	0.00	3	10.71	25	89.29	28	100.00	0.89	kept	0.7857143
iii	Multiple method used to commit suicide (more than 2)	0	0.00	3	10.71	25	89.29	28	100.00	0.89	kept	0.7857143
e	Cause of Death											
i	Due to mechanical asphyxia	1	3.57	2	7.14	25	89.29	28	100.00	0.89	kept	0.7857143
ii	Due to burn injury	0	0.00	3	10.71	25	89.29	28	100.00	0.89	kept	0.7857143
iii	Due to trauma/ blunt injuries	0	0.00	3	10.71	25	89.29	28	100.00	0.89	kept	0.7857143
iv	Due to electrocution injury	0	0.00	3	10.71	25	89.29	28	100.00	0.89	kept	0.7857143
v	Due to firearm injury	1	3.57	2	7.14	25	89.29	28	100.00	0.89	kept	0.7857143
vi	Due to poisoning by any route of administration	1	3.57	1	3.57	26	92.86	28	100.00	0.93	kept	0.8571429
vii	Due to overdose of drugs (by schedule drug listed in NDPS Act, 1985)	1	3.57	1	3.57	26	92.86	28	100.00	0.93	kept	0.8571429
viii	Due to overdose of drug (other than schedule drug listed in NDPS Act, 1985)	2	7.14	1	3.57	25	89.29	28	100.00	0.89	kept	0.7857143
ix	Due to secondary complication of any of the above method used for suicide	1	3.57	2	7.14	25	89.29	28	100.00	0.89	kept	0.7857143
x	Other, specify…..	1	3.57	4	14.29	23	82.14	28	100.00	0.82	kept	0.6428571

A total of 22 data elements (Table 2) (eight data elements from the first Delphi round and 14 from expert feedback/suggestions) were included in the second round of Delphi. In this round, seven out 22 data elements did not qualify the set norms of I-CVI and CVR, hence were excluded (Table 3). Quantitative analysis of the data elements was done by calculating the I-CVI and CVR in both rounds.

**Table 2:** Showing unqualified extracted data elements and other elements received through feedback during first round of Delphi Acronyms: Content Validity Index = I-CVI, Content Validity Ratio = CVR

Sr No	Unqualified data elements from 1^st^ round and Feedback questions	Disagree (1+2)		Neutral (3)		Agree (4+5)		Total number of responses & percentage		I-CVI - Round 2	Final Decision	CVR – Round 2
		count	%	count	%	count	%	count	%			
1	Gender [Any other (conversion of gender)]	1	3.57	2	7.14	25	89.29	28	100.00	0.89	kept	0.785714
2	Gender [Don't want to disclose]	6	21.43	9	32.14	13	46.43	28	100.00	0.46	discarded	-0.07143
3	Marital Relationship- Not stated	8	28.57	5	17.86	15	53.57	28	100.00	0.54	discarded	0.071429
4	Residence, Type of residence - unknown	3	10.71	6	21.43	19	67.86	28	100.00	0.68	discarded	0.357143
5	Residence, Type of residence - Other, specify	2	7.143	7	25	19	67.86	28	100.00	0.68	discarded	0.357143
6	Deceased residing with - unknown	3	10.71	5	17.86	20	71.43	28	100.00	0.71	discarded	0.428571
7	Deceased residing with - Other, specify	0	0	4	14.29	24	85.71	28	100.00	0.86	kept	0.714286
8	Religion - don’t want to disclose	8	28.57	4	14.29	16	57.14	28	100.00	0.57	discarded	0.142857
9	Determination of age of a deceased by using traditional methods (as suggested by few experts)	2	7.14	1	3.57	25	89.29	28	100.00	0.89	kept	0.785714
10	Who last saw him alive?	0	0.00	3	10.71	25	89.29	28	100.00	0.89	kept	0.785714
11	How was his behaviour before committing suicide?	0	0.00	3	10.71	25	89.29	28	100.00	0.89	kept	0.785714
12	Does victim (not deceased) reached out for help before committing the suicide?	1	3.70	1	3.70	25	92.59	27	100.00	0.93	kept	0.785714
13	Is there any abettor or factors abetting suicide of the victim?	0	0.00	3	10.71	25	89.29	28	100.00	0.89	kept	0.785714
14	History of suicide by 1st degree family member?	1	3.57	2	7.14	25	89.29	28	100.00	0.89	kept	0.785714
15	Monetary/Financial loss due to unpaid loan by parents?	0	0.00	1	3.57	27	96.43	28	100.00	0.96	kept	0.928571
16	Monetary/Financial loss due to farming or gambling?	0	0.00	2	7.14	26	92.86	28	100.00	0.93	kept	0.857143
17	Monetary/Financial loss in share market?	1	3.57	1	3.57	26	92.86	28	100.00	0.93	kept	0.857143
18	History of financial loss or any crime related to cybercrime or digital arrest.	0	0.00	1	3.57	27	96.43	28	100.00	0.96	kept	0.928571
19	History of excessive usage or exposure to social media or online gaming habit?	1	3.57	2	7.14	25	89.29	28	100.00	0.89	kept	0.785714
20	Addiction to smokeless tobacco?	1	3.57	1	3.57	26	92.86	28	100.00	0.93	kept	0.857143
21	Dyadic suicide death?	0	0.00	6	21.43	22	78.57	28	100.00	0.79	kept	0.571429
22	Suicidal Strangulation?	6	21.43	5	17.86	17	60.71	28	100.00	0.61	discarded	0.214286

**Table 3:** Excluded data elements after Delphi rounds Acronyms: Content Validity Index = I-CVI, Content Validity Ratio = CVR

Sr No	Unqualified Data Elements from 1st round & Feedback questions	Disagree (1+2)		Neutral (3)		Agree (4+5)		Total number of responses & percentage		I-CVI - Round 2	Final Decision	CVR
		count	%	count	%	count	%	count	%			
1	Gender [Don't want to disclose]	6	21.43	9	32.14	13	46.43	28	100.00	0.46	discarded	-0.07143
2	Marital Relationship- Not stated	8	28.571429	5	17.857143	15	53.571429	28	100.00	0.54	discarded	0.071429
3	Residence, Type of residence - unknown	3	10.714286	6	21.428571	19	67.857143	28	100.00	0.68	discarded	0.357143
4	Residence, Type of residence - Other, specify	2	7.1428571	7	25	19	67.857143	28	100.00	0.68	discarded	0.357143
5	Deceased residing with - unknown	3	10.714286	5	17.857143	20	71.428571	28	100.00	0.71	discarded	0.428571
6	Religion - don’t want to disclose	8	28.571429	4	14.285714	16	57.142857	28	100.00	0.57	discarded	0.142857
7	Suicidal Strangulation?	6	21.43	5	17.86	17	60.71	28	100.00	0.61	discarded	0.214286

Finally, after both Delphi phases and the calculation of I-CVI and CVR, the data elements that met the criteria were retained and included in the final MDS. Data elements that did not meet the set criteria were excluded from the final MDS for the suicide registry.

## Discussion

In this study, we developed an MDS comprising eight categories, with 169 data elements evaluated and approved for recording suicidal behaviors and associated causes of death following post-mortem examinations. The Ministry of Health and Family Welfare's (MoHFW) National Suicide Prevention Strategy identifies four priority areas for strategic suicide prevention, one of which is "strengthen surveillance and evidence collection" [10]. This MDS is comprehensive and suitable for collecting scientifically evidence-based suicide data to strengthen the suicide surveillance system. According to the authors, no prior attempt has been made to develop an MDS for a suicide registry in India, resulting in the absence of a national-level suicide registry [15]. The adoption and implementation of this MDS will benefit not only local and higher-level investigative agencies like the NCRB, but also the MoHFW's National Suicide Prevention Strategy (NSPS) in analyzing suicide cases and informing health-related policy decisions [25,26].

The standardized MDS will assist in identifying differences among suicide victims based on demographic, cultural, migration status and environmental factors across all Indian states [7,27]. This will help identify numerous risk factors contributing to suicide, particularly among hard-to-reach groups, such as migrant populations [28]. In this context, large-scale data collection will be enhanced by reducing errors at the medical or investigative (police) level through medico-legal post-mortem examinations, enabling accurate identification and categorization of suicide cases via the implementation of MDS in the Department of Forensic Medicine and Toxicology of the Medical Institute [29]. The gathered data using MDS will be accessible to policymakers, public health experts, clinicians, and law enforcement agencies for designing the suicide prevention strategies at local, state and national levels.

The strength of this study lies in the broad expertise of specialists from various disciplines and from different geographical areas/states of India, who provided valuable input in developing the MDS for suicide prevention, including data elements not previously identified in the literature. Several standardized data elements, such as marital/relationship status, the modified Kuppuswamy scale (for occupation, education, monthly income and socio-economic class), residential status (housing rental allowance (HRA)), family structure, elements evaluating the reason behind the suicide, family and social issues, clinical and psychological factors, and methods of suicide by self-harm, were validated by experts during the preparation and standardization of this MDS [17,30-32]. This standardized dataset enables more consistent data collection, analysis, and integration for suicide prevention.

As a continuation of this project, the MDS form will be converted into a web-based data application and deployed at four to five different institutions for data collection, accompanied by a user training manual. This training can be conducted online using web-based tools, making it more cost-effective, as the users are already trained health professionals, such as forensic medicine or mental health experts. The MDS will be updated as necessary based on additional feedback regarding its practicality, usability, implementation, and real-time data synchronization. However, it is also important to acknowledge the study’s limitations, including the small number of experts involved, which could be addressed in future studies by providing incentives for experts’ valuable contributions. In this study, all specialists voluntarily contributed to the study providing valuable inputs, without any compensation. Future research should also explore interoperability and adopt diverse approaches across various specialties to address any technical demands that may arise.

## Conclusions

This study developed a standardized MDS that offers the advantage of collecting scientifically evidence-based data related to suicide. This will help identify various risk factors contributing to suicide and address existing gaps influenced by demographic, cultural, migratory, and environmental differences across all Indian states. Based on this scientific data, multiple stakeholders - including physicians, healthcare workers, investigative agencies, and lawmakers - will be better equipped to implement effective suicide prevention measures.

## Appendices

**Table 4:** Questionnaires used during Delphi round One

Development and pilot testing of a minimum data set (MDS) to register the death by suicide in a tertiary health care setting		
Data Elements used during Delphi round - 1		
* Indicates required question		
Sr No	Heading	Data Elements
1	Expert’s Details	
1	Expert’s Email id*	
2	Full Name	
3	Expertise*	a) Community & family Medicine b) Forensic Medicine & Toxicology c) Intensivist d) Medicine e) Pediatrics f) Psychiatry g) Psychology h) Surgery
4	Professional experience of the expert*	a) Less than 5 years b) more than 5years but less than 10years c) more than 10 years
2	Deceased Profile Dear expert, kindly rate the data element which you think is to be included into this future surveillance for suicides. Please provide any feedback if you have particularly about inclusion or exclusion of any data elements. Please click if your answer is: 1 = Strongly disagree, 2 = Disagree, 3 = Neutral, 4 = Agree, 5 = Strongly agree	
1	Gender *	a) Male b) Female c) Transgender d) Any other (conversion of gender) e) Don't want to disclose
2	Age *	a) Based upon date of birth (DOB, if known) b) If DOB not known, then as reported on any document c) If DOB is not known or no document available, as informed by relative or police
3	Any feedback or suggestion from experts on deceased profile	
3	Religion, Relationship and Residence Status Dear expert, kindly rate the data element which you think is to be included into this future surveillance for suicides. Please provide any feedback if you have particularly about inclusion or exclusion of any data elements. Please click if your answer is: 1 = Strongly disagree, 2 = Disagree, 3 = Neutral, 4 = Agree, 5 = Strongly agree	
1	Religion*	a) Hindu b) Muslim c) Christian d) Sikh e) Buddhist f) Jain g) Any other religion h) Don't want to disclose
2	Marital/ Relationship Status *	a) Never married b) Widowed c) Divorced d) Separated e) Married f) Not stated g) Live in relationship (emotional relationship)
3	Family structure*	a) Proton (single individual) b) Electron (no married couple) c) Nuclear (single married couple with/without their unmarried children) d) Atom (nuclear family with any other family member (s) but no other married couple) e) Molecular (Exactly two married couples of any different generations (vertical levels) with/without unmarried people of any other generation) f) Joint (Two or more married couples of a single generation (horizontal level) or three or more couples if multiple generations (vertical levels) g) Quasi- (The prefix ‘‘quasi-” can be added to any of the previous types (III onward), for a couple who are sharing kitchen and financial resources as a married couple but not legally married)
4	Residence	
1	Residence (Based upon HRA), based upon “Revised List of Classification Cities for HRA of central government employees”. Govt. Employees India. Archived from the original on 25 AUGUST 2016. Retrieved 12 June 2015. *	a) X category b) Y category c) Z category
2	Residence (Based upon Population) *	a) Rural (up to 9999) b) Semi-urban (10,000 to 99,999) c) Urban (1,00,000 to 9,99,999) d) Metropolitan (10,00,000 & above)
3	Type of residence*	a) House/ Town home b) Apartment c) Homeless d) Treatment Facility (eg. for drug abuser, old age home) e) Correctional facility (eg. jail, prison, other detention facility) f) Unknown g) Other, specify….
4	Deceased Residing with*	a) Parents b) Spouse c) Roommate (s) d) Children e) No one resided alone f) Unknown g) Other, specify…
5	Recent Resident Problem*	a) Recent eviction b) Threat of eviction c) Recent foreclosure d) Threat of foreclosure
6	Any feedback or suggestion from experts on religion, relationship and residence status of deceased	
4	Socio-economic status as per Modified Kuppuswamy scale Dear expert, kindly rate the data element which you think is to be included into this future surveillance for suicides. Please provide any feedback if you have particularly about inclusion or exclusion of any data elements. Please click if your answer is: 1 = Strongly disagree, 2 = Disagree, 3 = Neutral, 4 = Agree, 5 = Strongly agree	
1	Occupation with score*	a) Professional (10) b) Semi- professional (06) c) Clerical (05) d) Skilled workers (04) e) Semiskilled workers (03) f) Unskilled workers (02) g) Unemployed (01)
2	Education with score*	a) Professional degree (07) b) Graduate or Post-graduate (06) c) Intermediate or post high school diploma (05) d) High school certificate (04) e) Middle school certificate (03) f) Primary school certificate (02) g) Illiterate (01)
3	Income with score*	a) >2,13,814 & above (12) b) 1,06,850 - 2,13,813 (10) c) 80,110 - 1,06,849 (06) d) 53,361 - 80,109 (04) e) 31,978 - 53,360 (03) f) 10,703 - 31,977(02) g) <10,702 (01)
4	Socio-economic class with score*	a) Upper class (26-29) b) Upper - middle (16 - 25) c) Lower - middle (11-15) d) Upper - lower (05-10) e) Lower (01 - 04)
5	Any feedback or suggestion from experts	
5	History obtained from investigating agency (Police/relative) Dear expert, kindly rate the data element which you think is to be included into this future surveillance for suicides. Please provide any feedback if you have particularly about inclusion or exclusion of any data element. Please click if your answer is: 1 = Strongly disagree, 2 = Disagree, 3 = Neutral, 4 = Agree, 5 = Strongly agree	
1	Who saw the body for the first time or found it?*	a) Family members - 1st degree b) Family members - other than 1st degree c) Friends d) Neighbours e) Police f) Social worker/NGO g) Others
3	Incidence site*	a) Own residence b) Rented house c) Forest area d) Farm e) At workplace f) Hostel g) School h) Police lock up i) Prison j) Construction site k) Old age home l) Other, please specify
4	Any feedback or suggestion from experts on use of socio-economic status as per Modified Kuppuswamy scale	
6	Elements to evaluate the reason behind the current suicide Dear expert, kindly rate the data element which you think is to be included into this future surveillance for suicides. Please provide any feedback if you have particularly about inclusion or exclusion of any data elements. Please click if your answer is: 1 = Strongly disagree, 2 = Disagree, 3 = Neutral, 4 = Agree, 5 = Strongly agree. Answer for first 7 question will be in format of Yes, No, Don't know.	
1	Is there any isolation by the deceased during the attempt? *	
2	Material or object used for suicide, is it already available? *	
3	If the answer for the above question is “No”, then is it purchased or brought from somewhere outside? *	
4	Is their suicide note found on the scene? *	
5	Is there any clear expression of suicide intention*	
6	Alleged purpose of suicide attempt*	
7	The number of suicidal ideation/attempts*	
8	History of Family issues*	a) Family conflict b) Peer conflict c) Spouse problems/ Marital-partner relationship difficulties d) Relationship breakdown with an intimate partner (past 1 month) e) Some legal issues or dispute f) Death of a close family member g) Parent separation h) Issue with Position in the household – head, child, wife, unknown
9	History of Social issues*	a) Social and teamwork activities b) Antisocial activities c) Work problems d) Recent job loss e) Issues related to Education like fail in exam, exam grade, suspension or ragging f) Victim of domestic violence
10	Any feedback or suggestion from experts on elements to evaluate the reason behind the current suicide	
7	Clinical and psychological factors Dear expert, kindly rate the data element which you think is to be included into this future surveillance for suicides. Please provide any feedback if you have particularly about inclusion or exclusion of anydata elements. Please click if your answer is: 1 = Strongly disagree, 2 = Disagree, 3 = Neutral, 4 = Agree, 5 = Strongly agree	
1	History of Medical Illness*	Diagnosed communicable illness such as HIV/TB/HBV/HCV Suffering from non-communicable illness, any chronic illness/condition (eg. cancer) Recent serious injury
2	History of mental disorders (eg. delusion, mood, personality & anxiety disorder, schizophrenia or any other disorder, sleep disorder) *	Answer for this question will be in format of Yes, No, Don't know.
3	History of Drug addiction*	a) Alcohol dependence b) Chronic smoker/Nicotine addiction c) Opioid dependence d) Cannabis dependence e) Any other drug dependence f) Any other drug dependence
4	Period during which suicide committed? *	a) 00.00am – 06.00Hrs b) 06.01hrs – 12.00Hrs c) 12.01Hrs – 18.00Hrs d) 18.01Hrs – 23.59Hrs
5	Any feedback or suggestion from experts on clinical and psychological factors	
8	Suicide Method Dear expert, kindly rate the data element which you think is to be included into this future surveillance for suicides. Please provide any feedback if you have particularly about inclusion or exclusion of any data elements. Please click if your answer is: 1 = Strongly disagree, 2 = Disagree, 3 = Neutral, 4 = Agree, 5 = Strongly agree	
1	Probable cause of death as reported by Police before PM examination*	a) Hanging b) Accidental Strangulation c) Suffocation d) Drowning e) Insecticide consumption f) Consumption of other poison/ poison which is not known g) Jumping (from height, in front of train or running vehicle) h) By setting fire with the use of inflammable agent i) By using Firearm weapon or by explosive material j) By using sharp object k) By using blunt object l) By use of electrocution m) Other, please specify
2	Suicide method - By self-harm*	a) Hanging/strangulation/ suffocation b) Drowning c) Pesticide (insecticide) consumption d) Consumption of other poison e) Jumping (from height, in front of train or running vehicle) f) Any other method which is not included above g) By setting fire with the use of inflammable agent h) By using Firearm weapon or by explosive material i) By using sharp object j) By using blunt object k) By use of electrocution l) Other, please specify
3	Suicide method - By self-poisoning*	a) Overdose of drug by oral ingestion b) Overdose of drug by iv/im/ by other routes c) Overdose of alcohol d) Exposure to organic solvents or hydrogenated hydrocarbons and their vapors e) Exposure to asphyxiant toxic gases f) Exposure to pesticide g) Exposure to unspecified chemicals or poison h) Others, please specify………
4	Number of suicide method used to commit suicide, which was confirmed after PME?*	a) Single method used b) Double method used c) Multiple method used to commit suicide (more than 2)
5	Cause of Death*	a) Due to mechanical asphyxia b) Due to burn injury c) Due to trauma/ blunt injuries d) Due to electrocution injury e) Due to firearm injury f) Due to poisoning by any route of administration g) Due to overdose of drugs (by schedule drug listed in NDPS Act, 1985) h) Due to overdose of drug (other than schedule drug listed in NDPS Act, 1985) i) Due to secondary complication of any of the above method used for suicide j) Other, specify………..
6	Any feedback or suggestion from experts on suicide method & cause of death	
Note: Rating for individual data elements of a question has been obtained from the experts.		

**Table 5:** Questionnaires used during Delphi round two

Development and pilot testing of a minimum data set (MDS) to register the death by suicide in a tertiary health care setting		
Data Elements used during Delphi round - 2		
Data Elements include - Elements which did not qualify set norms during Delphi round 1 & elements received via feedback from experts during Delphi round 2		
* Indicates required question		
Dear experts, kindly rate the data parameters which you think is to be included into this future surveillance for suicides and in this stage, we won't require any new feedback from expertise. 1 = Strongly disagree, 2 = Disagree, 3 = Neutral, 4 = Agree, 5 = Strongly		
Sr No	Parameters	
1	Gender [Any other (conversion of gender)] *	
2	Gender [Don't want to disclose] *	
3	Marital Relationship- Not stated *	
4	Residence, Type of residence - unknown *	
5	Residence, Type of residence - Other, specify *	
6	Deceased residing with - unknown *	
7	Deceased residing with - Other, specify *	
8	Religion - don’t want to disclose *	
9	Determination of age of a deceased by using traditional Forensic methods (as suggested by few experts) *	
10	Who last saw him alive? *	
11	How was his behavior before committing suicide? *	
12	Does victim (not deceased) reached out for help before committing the suicide? *	
13	Is there any abettor or factors abetting suicide of the victim? *	
14	History of suicide by 1st degree family member? *	
15	Monetary/Financial loss due to unpaid loans by parents? *	
16	Monetary/Financial loss due to farming or gambling? *	
17	Monetary/Financial loss in share market? *	
18	History of financial loss or any crime related to cybercrime or digital arrest. *	
19	History of excessive usage or exposure to social media or online gaming habit? *	
20	Addiction to smokeless tobacco? *	
21	Dyadic suicide death? *	
22	Suicidal Strangulation? *	
Note: Rating for individual data elements of a question has been obtained from the experts.		
